# Reproducible RNA Preparation from Sugarcane and Citrus for Functional Genomic Applications

**DOI:** 10.1155/2009/765367

**Published:** 2010-01-27

**Authors:** Mona B. Damaj, Phillip D. Beremand, Marco T. Buenrostro-Nava, Beth Riedel, Joe J. Molina, Siva P. Kumpatla, Terry L. Thomas, T. Erik Mirkov

**Affiliations:** ^1^Department of Plant Pathology and Microbiology, Texas AgriLife Research, Texas A&M System, Weslaco, TX 78596, USA; ^2^Laboratory for Functional Genomics, Department of Biology, Texas A&M University, College Station, TX 77843-3258, USA; ^3^Department of Trait Genetics and Technologies, Dow AgroSciences LLC, 9330 Zionsville Road, Indianapolis, IN 46268, USA

## Abstract

High-throughput functional genomic procedures depend on the quality of the RNA used. Copurifying molecules can negatively impact the functionality of some plant RNA preparations employed in these procedures. We present a simplified, rapid, and scalable SDS/phenol-based method that provides the high-quantity and -quality RNA required by the newly emerging biotechnology applications. The method is applied to isolating RNA from tissues of two biotechnologically important crop plants, sugarcane and citrus, which provide a challenge due to the presence of fiber, polysaccharides, or secondary metabolites. The RNA isolated by this method is suitable for several downstream applications including northern blot hybridization, microarray analysis, and quantitative RT-PCR. This method has been used in a diverse range of projects ranging from screening plant lines overexpressing mammalian genes to analyzing plant responses to viral infection and defense signaling molecules.

## 1. Introduction

Preparation of high-quality RNA is critical for functional genomic studies. Isolating high-quality RNA from biotechnologically important crops such as sugarcane and citrus is complicated by the presence of high concentrations of intrinsic polysaccharides, polyphenols, and other secondary metabolites. Levels of these compounds increase in plants under biotic and abiotic stresses, such as pathogen infection or drought [1, 2]. These metabolites tend to copurify with the RNA, interfering with downstream applications that are highly sensitive such as sequence expressed tag-marker-assisted polymorphism, cDNA library construction, and microarray hybridization. In addition, variability in purity from sample to sample will impact physiological and biochemical studies [3]. 

Several methods exist for isolating RNA from tissues of species with a high content of polysaccharides or polyphenols. These methods mainly use denaturing agents such as guanidine- and phenol-based extraction buffers in combination with isopropyl alcohol precipitation [4–6], detergents such as sodium dodecyl sulfate (SDS) [7] or cetyltrimethylammonium bromide (CTAB) [8, 9], followed by lithium chloride (LiCl) precipitations [8, 9]. Some improved methods combine guanidine and CTAB [10] or SDS and phenol [11, 12], with additional use of the antioxidant polyvinylpyrrolidone (PVP) [13] or benzyl chloride for cell wall degradation [14] during extraction. Other methods include an additional precipitation step using ethanol or 2-buthoxyethanol [15, 16]. Although these procedures produce high-quality RNA from specific species, most of them are time consuming or result in low yield. Thus, there is a need to improve methods for problematic plant species to increase speed of RNA preparation and provide both high quality and high quantities of RNA required by the new high-throughput biotechnological applications. 

We have developed a simple, rapid, and scalable procedure for isolation of high-quality RNA from sugarcane and citrus to facilitate the application of functional genomic studies in these crops. The procedure is a simplified SDS/phenol extraction method with sequential steps of purification from polysaccharides and polyphenols using 2-mercaptoethanol/PVP-binding, chloroform partitioning, and sodium acetate/ethanol- and LiCl–mediated precipitations. It relies on two extraction steps using automated homogenization from small amounts of tissue and extraction buffer and on two rounds of precipitation. In citrus, only one extraction step is needed. High yields and quality of RNA are consistently obtained from multiple samples. Low and high molecular weight-RNA as well as low- and high-abundant RNA isoforms can be recovered. 

The simplified RNA isolation method was compared with other RNA extraction methods used for functional genomic studies in sugarcane and citrus, namely, those based on guanidine thiocyanate [17–21], TRIZOL reagent (phenol and guanidine isothiocyanate) [22–26], and SDS/phenol [27–29]. The present study illustrates that the use of this method considerably accelerates the screening of transgenic plants containing high amounts of polysaccharides and secondary metabolites as well as the transcriptome analysis of genetically complex crops such as sugarcane in response to stress. 

## 2. Material and Methods

### 2.1. Genetic Constructs and Plant Transformation

Constructs carrying a synthetic mammalian gene (0.455 kilobase [kb]), codon optimized for expression in either monocots or dicots, were generated. The sugarcane construct consisted of the mammalian gene cloned into the *Bam*HI-digested vector-pZero (Invitrogen Life Technologies, Carlsbad, CA) and placed under the control of the maize *ubiquitin 1* (*Ubi1*) promoter and the 35S cauliflower mosaic virus terminator, yielding pZero*Ubi1*:mammalian gene. The construct for citrus transformation was generated by cloning the mammalian gene into the binary vector pBIN34S [30] to produce pBIN34S:mammalian gene. 

Embryogenic callus was established from young leaf bases and immature flowers of commercial sugarcane (*Saccharum* spp. hybrid, cultivar CP72-1210) and transformed with pZero*Ubi1*:mammalian gene together with the *Ubi1*:*BAR*-pUC8 (pAHC20) plasmid [31] as described previously [32, 33]. RNA was isolated from leaves of 4-month-old control and transformed plants for northern blot analysis. Citrus transformation with pBIN34S:mammalian gene was carried out using seedling-derived epicotyl segments of the Hamlin orange cultivar (*Citrus sinensis* Pers.) and *Agrobacterium tumefaciens* strain EHA105 [30, 34]. 

The presence and expression of the mammalian gene was confirmed on both citrus and sugarcane plants by Southern (data not shown) and northern blot analyses. Leaf tissues from 3- to 4-month-old control and transformed plants, grown in a controlled-environment greenhouse (28°C with 14-hour-light/10-hour-dark), were used for northern blot analysis.

### 2.2. Plant Growth and Treatment Conditions

Sugarcane (*Saccharum *spp. hybrid, cultivar CP72-1210) was grown in potting mix (Redi-earth mix, Scotts, Hope, AR) in a controlled-environment greenhouse (28°C with 14-hour-light/10-hour-dark) for stress-induction experiments. Four-month-old plants were treated with the stress-regulated hormones, salicylic acid (SA) or methyl jasmonate (MeJA) (Sigma-Aldrich, Saint Louis, MO). Treatments were conducted by spraying plants till run-off with a 5 mM SA solution in water and 0.05% (v/v) Tween-20 and kept at 80% humidity in the greenhouse. MeJA (a volatile form of jasmonates) treatment was carried out by placing a cotton swab containing 500 *μ*L of 100 *μ*M solution in 0.1% (v/v) ethanol and 0.05% (v/v) Tween-20 at the soil surface, close to the main stem of plants kept in clear plastic bags inside the greenhouse. Control plants were treated identically except without the addition of SA or MeJA. Stems, leaves, and roots of treated and control plants were collected at 0, 24, and 48 hours of treatment. At least three plants were tested for each time point of treatment and pooled to produce a biological sample. Two biological replicates were used. 

Viral infection of sugarcane was carried out by inoculating with sap extract from sorghum (*Sorghum bicolor* cultivar Rio) infected with a compatible strain of *Sorghum mosaic virus* (SrMV) (described by Yang and Mirkov [35]) according to Ingelbrecht et al. [33]. Control plants were not inoculated but were otherwise treated identically. Leaf RNA from six SrMV-infected sugarcane plants was used for the microarray analysis.

### 2.3. RNA Extraction Protocol

A simple scalable protocol (TENS-PCI) was developed for the isolation of RNA from sugarcane and citrus tissues. The protocol steps are outlined in Table 1. 

#### 2.3.1. Isolation of RNA by the TENS-PCI Method

**Extraction.**

For small-scale RNA isolation, tissue (0.1-0.2 g, snap frozen in liquid nitrogen) was homogenized in 2 mL screw-cap microcentrifuge tubes for 30 seconds at 5000 rpm with the Precellys 24 homogenizer (MO BIO Laboratories, Carlsbad, CA) in the presence of a ceramic spherical bead (0.64 cm-diameter). Up to 24 samples could be processed at a time with this homogenizer. A mixture (600 *μ*L total volume) containing equal volumes of TENS extraction buffer and phenol (pH 4.3): chloroform: isoamyl alcohol (1.0: 0.8: 0.2) (PCI) reagent was added to the homogenate with thorough mixing by hand. The TENS buffer consisted of 10 mM hydroxymethyl aminomethane (Tris-HCl) (pH 7.5), 1 mM ethylenedinitrilo-tetraacetic acid (EDTA) (pH 8.0), 0.1 M sodium chloride (NaCl), 1% (w/v) sodium dodecyl sulfate (SDS), 2% (w/v) polyvinylpyrrolidone (PVP)-40, and 7% (v/v) 2-mercaptoethanol. The homogenate was centrifuged at 10,000 *g *for 20 minutes at 4°C, and the supernatant was re-extracted with an equal volume of PCI. In citrus, only one extraction (TENS/PCI) was performed.

**Precipitation.**

The supernatant was mixed with 0.1x volume of 3 M sodium acetate (NaOAc) (pH 5.2) and 3.0x volume of ice-cold 100% (v/v) ethanol and incubated at −20°C for 1 hour. The precipitate was collected by centrifugation at 10,000 g for 20 minutes at 4°C, air-dried briefly, and dissolved in 100 *μ*L of nuclease-free water. A second round of precipitation was performed, using an equal volume of 4 M LiCl (100 *μ*L) at −20°C overnight. The RNA pellet was recovered by centrifugation at 10,000 g for 20 minutes at 4°C, washed with 70% (v/v) ethanol, air-dried, and dissolved in 50–100 *μ*L of nuclease-free water.

Large-scale RNA preparations from 60 g of stem tissue, 1 g of leaf, or 1 g of root tissue followed the same protocol as above by proportionally scaling up the volumes of the buffers. 

Total RNA was treated with RNase-free DNase I (Applied Biosystems/Ambion, Austin, TX) prior to use.

#### 2.3.2. Comparing the TENS-PCI Method with Standard RNA Isolation Methods

The TRIZOL reagent (phenol and guanidine isothiocyanate [36]) (Invitrogen Life Technologies) as well as two standard commercial laboratory kits, the RNeasy Plant Mini kit (Qiagen, Valencia, CA) that uses guanidine isothiocyanate or guanidine hydrochloride, and the FastRNA Pro Green kit (MP Biomedicals, LLC, Solon, OH), which is phenol-based, were tested in parallel with the TENS-PCI method for RNA extraction.

### 2.4. RNA Yield and Integrity

RNA was quantified using a NanoDrop 1000 spectrophotometer (Thermo Fisher Scientific, Wilmington, DE). RNA integrity (size and distribution of the extracted RNA molecules) was determined using an Agilent 2100 Bioanalyzer with the RNA 6000 Nano LabChip kit (Agilent Technologies, Palo Alto, CA). The ratio of the peak areas of the plant large subunit (LS) (about 25S) to the small subunit (SS) (18S) ribosomal (r) RNAs was assessed by the Bioanalyzer software.

### 2.5. RNA Amplification

Amplified RNA (aRNA) was prepared from sugarcane total RNA by the Eberwine method [37] using a MessageAmp aRNA kit (Applied Biosystems/Ambion) following the manufacturer's instructions. One to five *μ*g of total RNA from sugarcane tissue was used for each amplification, and 50–100 *μ*g of aRNA was obtained after one round of amplification.

### 2.6. Northern Blot Analysis

Total RNA (10 *μ*g each lane) from leaf tissues of sugarcane or citrus lines transgenic for the mammalian gene was fractionated on denaturing formaldehyde agarose gel and blotted onto nylon membranes (Nytran^R^ SuperCharge, Schleicher and Schuell BioScience, Inc., Keene, NH) in 10x SSPE buffer [38]. RNA blots were hybridized with probes amplified by PCR using primers derived from the full-length coding region of the target gene. PCR products were labeled with [^32^P]*α*-dATP by random priming using Klenow Exo^−^ DNA polymerase (New England BioLabs Inc., Ipswich, MA). Hybridization and washes were conducted in accordance with the method of Church and Gilbert [39]. Hybridized blots were visualized and quantified with the BAS-5000 scanning system (Fujifilm Life Science USA, Stamford, CT). RNA loading and transfer efficiency was normalized relative to the band intensity of the sugarcane constitutive *ubiquitin *gene [40].

### 2.7. cDNA Microarray Preparation and Analysis

A set of 229 stem-expressed cDNA clones were initially identified by the differential hybridization of a sugarcane stem cDNA library (13,824 clones) [40] and used to construct a cDNA microarray. The identity of the arrayed clones was confirmed by cycle sequencing and Blastx search. Information on the nucleotide sequence of these clones can be found at http://enterprise.bio.tamu.edu/. cDNA inserts in the pCR2.1 vector (Invitrogen Life Technologies) were amplified and printed on PL-100C poly-L-lysine-coated glass slides (CEL Associates, Pearland, TX) as described [41]. 

Randomly primed fluorescent probes were produced from aRNA samples using the 3DNA Array 350RP expression array detection kit (Genisphere, Hatfield, PA) as recommended. The fluorescent dye on probes derived from the experimental aRNA was Cy5, whereas the dye on control probes was Cy3. Hybridizations and washings followed Genisphere's suggestions. 

Labeled arrays were scanned with an Affymetrix 428 array scanner (Affymetrix, Santa Clara, CA). Resulting images were analyzed with GenePixPro (Axon Instruments, Union City, CA). Data files were further analyzed using GeneSpring (Agilent Technologies) to facilitate normalization, parameter assignment, and filtering. Experimental values were divided by the control values and further normalized relative to the positive control genes: *glutathione-S-transferase*, *G protein-coupled receptor*, *histone deacetylase*, *ribulose epimerase*, *tubulin,* and *ubiquitin* [40]. Regulated genes were defined as those with a two-fold or higher amplitude change in their normalized ratios and a *t*-test *P*-value of .05 or less. Two biological samples were used for each tissue type or time point. Each biological sample was used for three hybridizations. Six microarray hybridizations were conducted per sample. A total of six microarray hybridizations with dye swaps were carried out per tissue or treatment.

### 2.8. Quantitative RT-PCR Analysis

First-strand cDNAs were synthesized from DNase I-treated aRNA (2 *μ*g) using the TaqMan^R^ reverse transcription kit (Applied Biosystems, Foster City, CA). qRT-PCR was performed on an ABI PRISM 7700 (Applied Biosystems) with the SYBR^R^ Green PCR master mix (Applied Biosystems) according to the manufacturer's instructions. Primers were designed with the Primer Express1.5 software (Applied Biosystems). qRT-PCR was performed twice in triplicate with two biological repeats of aRNA. Results were analyzed with SDS1.7 software (Applied Biosystems) and recorded as *C*_*T*_ (threshold cycle) values. Each transcript was quantified relative to that of the sugarcane *ubiquitin *gene, using the comparative *C*_*T*_ method (user bulletin 2, ABI Prism 7700 sequence detection system; Applied Biosystems). A threshold of a two-fold difference in relative mRNA levels was used to designate genes as induced in one tissue type or one treatment time. Primer pairs used for some representative genes were as follows: *OMT* (5′-agattcggcaagctcttcgac-3′ [F] and 5′-ttgccacgatgtccatgatg-3′ [R]), *DIR1* (5′-cattcggcaaaacaacagaca-3′ [F] and 5′-gcgtccaaagaaacagatga-3′ [R]), *DIR11* (5′-atcaatcaagcacaatataa-3′ [F] and 5′-agatcgtgaaaagatacatt-3′ [R]), *DIR12* (5′-gcttgatcgactagcgc-3′ [F] and 5′-gcacaagaagcagctg-3′ [R]), *DIR16* (5′-cctgggcgcttctaccaac-3′ [F] and 5′-acacttgtcgatcaagcgtcg-3′ [R]), and *ubiquitin* (5′-ccaaaccccgacgatcc-3′ [F] and 5′-tctcgtacttgtgccggtcc-3′ [R]).

## 3. Results and Discussion

We are interested in carrying out functional genomic studies on sugarcane and citrus, two biotechnologically important crops that are rich in fiber, polysaccharides, and secondary metabolites. The handful of such studies with these crops employed RNA extracted using TRIZOL reagent [22–26], guanidine thiocyanate [17–21], or SDS/phenol [27–29]. We tested the TRIZOL reagent, the standard SDS/phenol method, as well as two commercially available RNA extraction kits, one based on guanidine (RNeasy) and the other on phenol (FastRNA Pro Green), to isolate RNA from sugarcane. We found that these methods did not perform well across multiple experiments. The RNA obtained produced a high background or a very weak signal when used in northern blot or microarray analysis. The major aim of the current study was to develop a simple, rapid, and scalable method that yields high-quantity and -quality RNA from sugarcane and citrus. 

### 3.1. Developing the TENS-PCI Method for Preparation of High-Quantity and -Quality RNA

The TENS-PCI method is a simplified RNA isolation technique that uses SDS and phenol with a high concentration of antioxidants (PVP-40 and 2-mercaptoethanol) for extraction and two rounds of precipitation to yield high-quantity and -quality RNA from 100 mg to 60 g of tissue in an efficient manner. The method is simplified in terms of requiring less manipulation, with only two extraction steps and two rounds of precipitation. It is also considered as a rapid isolation method due to the reduced time needed for automated tissue homogenization (30 seconds) and RNA precipitation (one precipitation for one hour and another for 16 hours). 

Optimization of the TENS-PCI micro-scale isolation method was achieved by reducing the leaf tissue weight from 1.0 g usually required for grasses [12] to 0.1 g. This is very similar to the amounts generally harvested from succulent tissues of dicot species. For the macro-scale method, only one gram of leaf or root tissue was used for extraction as opposed to 4 g of sugarcane leaf roll used in the SDS-phenol method of Carson and Botha [27, 28]. 

Automated tissue homogenization allowed the extraction of 24 samples in a very short period of time (30 seconds) in small tubes (2 mL), using a reduced extraction volume (600 *μ*L). Higher-throughput preparations could be easily achieved with this method by adapting the use of a higher-capacity homogenizer. 

An important component of the TENS-PCI extraction buffer was the presence of high levels of NaCl (0.1 M) used to increase the solubility of polysaccharides, reducing their coprecipitation with RNA in subsequent steps [42]. Higher concentrations of the antidoxidant 2-mercaptoethanol (7%), together with PVP (2%), were also used into the extraction buffer, as compared to earlier methods (0% to 5% 2-mercaptoethanol or 0% to 2% PVP) in woody plants and grasses [6, 10, 12, 16, 27]. This was done to improve the sequestering of phenolic compounds that are released during tissue homogenization [43, 44]. 

Two precipitation steps were included in the TENS-PCI method as opposed to the SDS-phenol method of Carson and Botha [27, 28] that used three rounds of precipitation, with two of them performed overnight. The first precipitation step of the TENS-PCI method, with NaOAc and ethanol for one hour, precipitates both nucleic acids and polysaccharides. The second, with a high-molarity LiCl solution (4 M) for 16 hours, differentially precipitates RNA from DNA and residual polysaccharides, thereby increasing RNA purity [45]. This significantly reduced the time needed to recover the RNA. 

Incorporation of high amounts of antioxidants (7% of 2-mercaptoethanol and 2% of PVP) into the TENS-PCI extraction buffer as well as the adoption of only two rounds of selective precipitation proved to be efficient in removing polysaccharides and phenols. The TENS-PCI method yielded RNA with low levels of polysaccharides/phenols as indicated by the spectrophotometric ratios of A_260_ : A_230_ that are close to 2.0 (Table 2). Ratio values of 1.8 to 2.0 are usually considered an acceptable indicator of high-purity RNA [38, 46]. However, the RNA isolated by the RNeasy-guanidine hydrochloride and the FastRNA Pro Green methods tested in this study displayed low A_260_ : A_230_ ratio averages of 1.20 and 1.17, respectively, (Table 2), suggesting high levels of contamination by polysaccharides and/or polyphenols. These results are in agreement with previous reports on the presence of polysaccharides/phenols in RNA extracted from grapevine [44], plum [44], lemon [44], and London plane tree [6] using the RNeasy, the guanidine, and the standard SDS/phenol and CTAB methods (Table 2). 

Previous reports showed that stress treatments such as pathogen infection or water and nutrient deficiencies can enhance the levels of accumulation of polysaccharides and secondary metabolites, particularly phenols, in plants [1, 2]. Our results indicate that the quality of RNA isolated by the TENS-PCI method was not affected by infection with the *Sorghum mosaic virus* (SrMV) pathogen or treatment with the stress-regulated hormones, salicylic acid (SA), and methyl jasmonate (MeJA). For example, average A_260_ : A_230_ ratios of 2.37 ± 0.12 and 1.98 ± 0.06 were obtained with RNA extracted with TENS-PCI from plants treated with MeJA for 24- and 48-hours, respectively, compared to those of RNA extracted from the control plants at 24- and 48-hour treatment with water/ethanol, which were 2.21 ± 0.11 and 2.25 ± 0.14, respectively. 

The TENS-PCI method was also the most efficient in removing proteins, with RNA samples from sugarcane and sweet orange repeatedly exhibiting A_260_ : A_280_ ratio values around 2.0 (Table 2). The phenol-based FastRNA Pro Green kit yielded a lower purity RNA, as detected by the slightly lower A_260_ : A_280_ ratio value, that is, 1.79 (Table 2). Low values were also reported for RNA extracted from London plane tree [6] with the SDS/phenol, guanidine, or CTAB method (Table 2). 

In the case of the RNeasy-guanidine isothiocyanate kit, acceptable A_260_ : A_230_ and A_260_ : A_280_ ratios were obtained, but yields were low (Table 2). This is similar to what has been reported for the rapid CTAB method in grapevine, plum, and lemon [44] (Table 2). An improvement in RNA yield was achieved with the TENS-PCI micro-scale method (5.2 *μ*g/mg tissue) by 4.7-fold in leaf as compared to the two tested kits, the RNeasy (0.11 *μ*g/mg tissue) and FastRNA Pro Green (0.11 *μ*g/mg tissue) (Table 2), although the quantity of the starting tissue was the same for all tested methods. The low RNA yield could be attributed to the presence of polysaccharides, saturating the binding capacity of the resin columns supplied with the kits during the RNA isolation step. A significant increase in the RNA yield was observed with the TENS-PCI micro-scale method compared to non-kit based RNA isolation methods that have been used for other woody plants. The increase in yield was on average of 368.8-fold for leaves and 366.7-fold for stems as opposed to the standard SDS/phenol, guanidine, and CTAB methods used for grapevine [44], lemon [44], plum [44], and London plane tree [6] (Table 2). 

We further assessed the performance of the TENS-PCI method across tissues and stress-related treatments, by checking the integrity of the RNA isolated from different sugarcane tissues such as leaf, stem and root, as well as from plants treated with the SrMV pathogen or the stress-regulators, SA and MeJA, using chip-based microcapillary electrophoresis. We similarly tested RNA obtained with the two kits, FastRNA Pro Green and RNAeasy Mini Plant (guanidine isothiocyanate). RNA degradation was evaluated using the ratios of the area under the curve of the peaks corresponding to the signal intensity of the large subunit to that of the small subunit (LS : SS) rRNA (based primarily on checking the quantity and quality of the ribosomal subunits). Values of LS : SS rRNA ratios of about 2.0 are considered to be indicators of good RNA integrity, depending on the tissue analyzed and the biological system used [47, 48]. 

**Figure 1:**
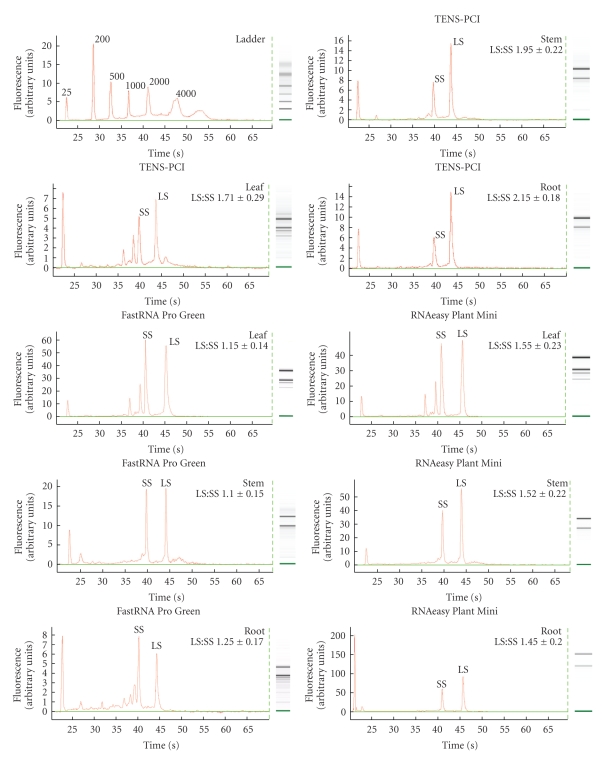
Assessment of the integrity of total RNA isolated by the TENS-PCI method, and the two kits, FastRNA Pro Green and RNeasy Plant Mini, from sugarcane leaf, stem, and root tissues, using chip-based electrophoretic separation with a bioanalyzer. A total of 100 ng of RNA was loaded per well. Representative RNA profiles corresponding to the three tissues are shown. Gel-like images of RNA from each tissue, generated from the RNA profiles, were included for clarity on the right side. The Agilent RNA 6000 Nano ladder is included for sizing. SS and LS represent the small and large ribosomal subunits, respectively. Numeric values represent mean and standard error of two biological replicates and at least three technical repeats.

**Figure 2:**
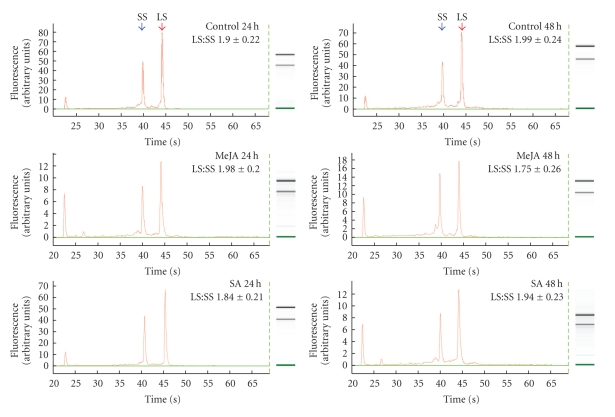
Assessment of the integrity of total RNA isolated by the TENS-PCI method from sugarcane stem tissues of plants treated with stress-regulated hormones, using chip-based electrophoretic separation with a bioanalyzer. A total of 100 ng of RNA was loaded per well. Representative RNA profiles corresponding to stem tissues of plants after treatment with SA (5 mM) or MeJA (100 *μ*M) at 0, 24, and 48 hours (h) are shown. Gel-like images of RNA from each treatment, generated from the RNA profiles, are included for clarity on the right side. SS and LS represent the small and large ribosomal subunits, respectively. Numeric values represent mean and standard error of two biological replicates and at least three technical repeats.

Elaborate measures designed to improve RNA purity can increase the risk of RNA that is degraded in the process. The simplified TENS-PCI method produced a high-purity intact RNA from leaves, stems, and roots, showing a non-significant variation among these tissues, as reflected by the ratios of the LS : SS rRNA peak areas of the RNA profiles of the tested samples (Figure 1). RNA of high integrity was also isolated from plants infected with the SrMV pathogen or treated with stress-regulated hormones. No significant RNA degradation occurred as shown by the intact rRNA peaks and the LS : SS ratios (Figure 2). The overall values across tissues and treatments for the LS : SS ratios ranged from 1.71 to 2.15 (Figures 1 and 2). 

Low-integrity RNA was obtained with the FastRNA Pro Green kit as shown by the low LS : SS ratios for the three tested tissues, leaf, root, and stem (Figure 1). The RNeasy-guanidine isothiocyanate yielded a better purity RNA, especially from stem and root, although the LS : SS ratio values were significantly lower than those obtained with the TENS-PCI method, indicating that RNA degradation had occurred (Figure 1). Thus, this method produced RNA of good purity but with a lower yield and lower quality than did the TENS-PCI (Table 2). 

### 3.2. RNA Isolated with the TENS-PCI Method is Suitable for Northern Blot Analysis

**Figure 3:**
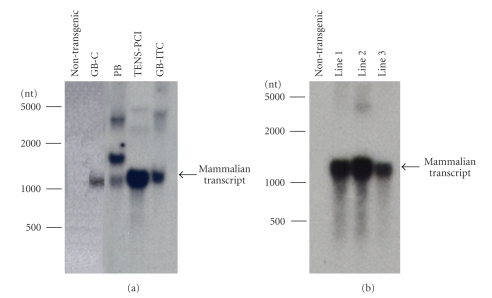
Northern analysis of the expression levels of sugarcane (a) and sweet orange (b) lines overexpressing a mammalian gene. Total RNA was isolated from leaf tissues by the TENS-PCI, the RNeasy guanidine-based (GB), and phenol-based (PB) methods. GB-ITC refers to guanidine isothiocyanate and GB-C to guanidine hydrochloride. RNA (10 *μ*g each lane) was fractionated, blotted, hybridized, washed, and imaged as described in Material and Methods.

Over 350 transgenic sugarcane and citrus plants were generated that constitutively expressed a mammalian gene. These were screened by Southern blot hybridization for presence and copy number of the gene (data not shown). To demonstrate the suitability of TENS-PCI for detecting specific transcripts, we tested the expression of the mammalian gene in leaves of transgenic sugarcane and citrus lines using northern blot hybridization. RNA isolated from the same lines using the RNeasy Plant Mini and the FastRNA Pro Green kits was also tested. Binding of the probe was the strongest and the most specific with RNA extracted by the TENS-PCI method, resulting in an intense band corresponding to the expected mammalian transcript size, of about 1.20 kb (Figures 3(a) and 3(b)), indicating that the RNA is intact and non-degraded. Binding of the mammalian probe to the RNA obtained with the RNeasy (GB-ICT and GB-H) and the FastGreen Pro (PB) methods was of low specificity since multiple bands were detected (Figure 3(a)). Furthermore, this binding was greatly inhibited (Figure 3(a)), probably due to the presence of secondary metabolites that were copurified with the RNA. This shows that the RNeasy and FastGreen Pro kits did not produce RNA adequate for northern blot hybridization of sugarcane and citrus. Previous studies have reported the inefficiency of these tested kits in extracting high-quality RNA from tissues rich in polysaccharides or polyphenols [13, 16, 44]. 

The inconsistency in RNA quality provided by the tested kits can impede the quantitation of the signal strength across multiple samples. Using the TENS-PCI micro-scale method, we were able to measure the transcriptional levels of multiple transgenic lines in a short time to identify high expressers of a mammalian gene. Our results were corroborated by Western analysis of protein levels and measurement of enzyme activity (data not shown). Thus, the TENS-PCI method represents a good option when limited amounts of tissue samples are available, since it consistently produces high-quantity and -quality RNA and does not interfere with the detection of specific transcripts.

### 3.3. RNA Extracted with the TENS-PCI Method Is Successfully Amplified for the Generation of Reproducible Microarray Data

Important sources of variability in genomic data include variations in tissue processing, RNA preparation and assay protocols, as well as inherent tissue heterogeneity [49, 50]. Of particular interest is the effect of the quality of the starting RNA. Jahn et al. [48] have observed that low-quality RNA samples did not show a significant difference in relative transcript expression ratios for a protein when RNA from mutants deficient in the gene for that protein was compared to RNA from the wild-type organism. This illustrates the importance of using high-quality RNA for reproducibly detecting significant differential gene expression data from transcriptome analyses. 

**Figure 4:**
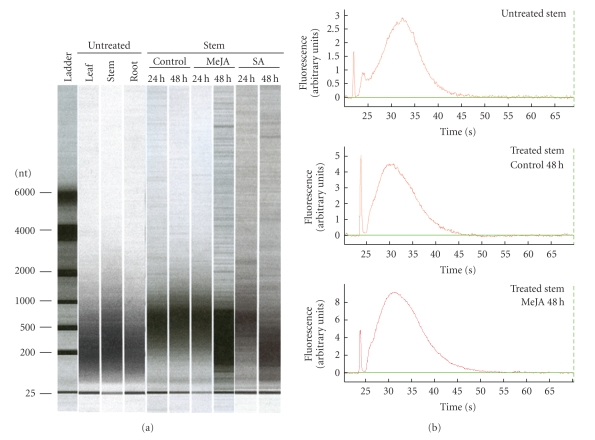
Chip-based electrophoretic separation of amplified total RNA (aRNA) isolated by the TENS-PCI method. aRNA was analyzed using an Agilent 2100 Bioanalyzer. A total of 20 ng of aRNA was loaded per well. (a) Densitometry simulation for aRNA from stem, leaf and root tissues of untreated plants as well as from stem tissues of plants after treatment with SA (5 mM) or MeJA (100 *μ*M) at 0, 24 and 48 hours (h). (b) Representative profiles of aRNA corresponding to stems of untreated plants, as well as to stems of control (mock: water and 0.1 % ethanol, 48 hours) and treated plants (MeJA at 48 hours). The Agilent RNA 6000 Nano ladder is included for sizing.

Feldman et al. [51] have described the advantages of amplifying RNA for improving microarray analysis. RNA amplification is dependent on the quality of the input RNA. The RNA obtained with the TENS-PCI method from different tissues and hormonal treatments was successfully amplified to provide sufficient amounts for high-throughput transcript profiling studies. The yield and size of the aRNA produced (Figure 4) were in the range expected from good-quality input RNA. This provides a functional proof of the quality of the RNA isolated by the TENS-PCI method. 

Microarray analysis performed with aRNA derived from the TENS-PCI method revealed significant differences in relative gene expression ratios, as reflected by the two-fold up- or down-regulation of the identified genes (Table 3, see Figure 1S in Supplementary Material available online at doi: 10.1155/2009/765367). No such differences were detected in the microarray data generated with aRNA derived from the RNeasy-guanidine isothiocyanate method (Supplementary Figure 1S). Furthermore, high correlation for the relative gene expression ratios was obtained among the replicate experiments performed with the TENS-PCI RNA (Pearson correlation coefficient [*r*] = 0.89–0.96; *t*-test *P* ≤.05) (Supplementary Figure 1S). Lower correlations (*r* = 0.65–0.70) were obtained from comparisons between replicate experiments that used RNA extracted with the RNeasy-guanidine isothiocyanate method (Supplementary Figure 1S). The detection of differential gene expression as well as the small variation between replicate experiments shows that the TENS-PCI RNA is of high quality to generate biologically significant and reproducible microarray data.

### 3.4. Defense- and Stress-Responsive Marker Genes Are Identified in the Transcript Profiling Analysis

Plant stress responses are largely mediated by phytohormones that trigger conserved defense mechanisms, with intricate signaling pathways leading to protection. Salicylic acid (SA) and the jasmonates (including methyl jasmonate (MeJA)) are major signaling molecules that regulate such protective responses via synergistic and antagonistic actions, referred to as signaling cross-talk [52–55]. To further demonstrate the functional quality of the TENS-PCI RNA, we performed a transcriptome analysis, looking specifically at defense- and stress-responsive genes. We did a focused microarray analysis to monitor the mRNA expression profiles of 229 sugarcane stem-regulated cDNAs in response to biotic and hormonal stresses. The cDNA microarray was hybridized to cDNA probes synthesized from RNA isolated by the TENS-PCI method from stem, leaf, and root tissues and from stem of plants treated with SA, or MeJA, or infected with the compatible pathogen *Sorghum mosaic virus* (SrMV). 

The micorarray analysis identified three major transcript profiling groups (Table 3) that consist of (a) stem-expressed genes that are co-induced by SA and MeJA, (b) genes that are induced by SA and repressed by MeJA, and (c) genes that are induced by MeJA and repressed by SA. The microarray data were confirmed by qRT-PCR analysis (Table 3). The genes in these groups are predominantly implicated in defense response, secondary metabolism, and fiber biosynthesis. They include the defense-related and fiber biosynthesis *dirigent* (*DIR*) family [17, 56] and *O*-*methyltransferase* (*OMT*) [57] genes as well as an antimicrobial *chitinase* gene [58] (Table 3). These results are consistent with earlier studies. Casu et al. [17] reported on the abundance of *DIR* genes in sugarcane maturing stems. MeJA has previously been shown to induce a number of *DIR* genes in sugarcane root [59]. *OMT* has been reported to be expressed in mature sugarcane stems [17]. Previous work has shown *OMT* to be induced in barley by MeJA [60] and in sorghum by SA and MeJA [61]. Cooperative regulation of chitinase by SA and MeJA has been observed in sorghum [61]. 

In addition, many of the genes that are regulated by SA and/or MeJA were two-fold down-regulated upon SrMV infection in our microarray analysis (Table 3). Such genes encode several DIR proteins and a chitinase. This down-regulation of gene expression upon viral infection is in agreement with Shi et al. [62, 63] who observed a differential expression of defense- and stress-responsive genes, including those coding for chitinases, in near-isogenic maize lines challenged with the *Sugarcane mosaic virus*. 

In summary, transcript profiling analysis of the sugarcane stem in response to the defense-inducing and stress-regulated hormones, SA and MeJA, has enabled the identification of marker genes that are associated with defense and stress responses. Such genes, specifically *OMT* and *DIRs*, were of particular interest to us in relation to their relevance as stem-regulated and stress-induced type markers reflecting both, the stem- and the stress-regulated origin of the RNA extracted by the TENS-PCI method.

## 4. Conclusions

We have developed a simple, rapid, and scalable protocol enabling an efficient and robust extraction of RNA from sugarcane and citrus on a micro- and macro-scale, reducing significantly the cost of RNA extraction per sample. Compared to other protocols, the presented TENS-PCI method is a simplified method that consists of two extraction steps for sugarcane and one for citrus, using SDS, phenol, and a high concentration of antioxidants (2-mercaptoethanol and polyvinylpyrrolidone-40) as well as two rounds of precipitation (sodium acetate/ethanol and lithium chloride). This method represents a good option since it combines the advantages of high RNA recovery (especially when limited amounts of tissue are available), high RNA integrity, reproducibility among biological and experimental replicates, and applicability to a wide range of tissues. We have demonstrated that this method accelerates the screening of transgenic plants with tissues rich in polysaccharides and secondary metabolites, using northern blot analysis. We have further shown that the high-quality RNA obtained by the TENS-PCI method can be easily amplified to generate reproducible and biologically significant gene expression data. We provide evidence of the utility of the RNA extracted by the TENS-PCI method in sensitive assays by showing that several defense- and stress-responsive marker genes are differentially regulated during the transcript profiling of part of the sugarcane transcriptome in response to pathogenic and hormonal stresses. These data corroborate with previously reported findings on the signaling pathways governing the plant stress response. We anticipate that the application of the TENS-PCI method in novel high-throughput functional genomic technologies such as next generation DNA sequencing will shed more light into the cross-talk signaling in biotechnologically important crops with complex genomes, such as sugarcane.

## Supplementary Material

Supplementary FIGURE 1S: Reproducibility of the data obtained from the microarray analysis of gene expression profiling of the sugarcane stem, using amplified RNA (aRNA).

## Figures and Tables

**Table 1:** The TENS-PCI protocol used for RNA isolation.

(1) Extraction	(a) TENS ^(a)^ and PCI ^(b)^ (equal volume)
	(b) PCI
(2) Precipitation	3 M sodium acetate (pH 5.2) (0.1x volume)
	Ethanol (3.0x volume)
(3) Re-precipitation	4 M lithium chloride

^(a)^ TENS: 10 mM hydroxymethyl aminomethane (Tris-HCl) (pH 7.5), 1 mM ethylenedinitrilo-tetraacetic acid (sodium EDTA) (pH 8.0), 0.1 M sodium chloride (NaCl), 1% (w/v) sodium dodecyl sulfate (SDS), 2% (w/v) polyvinylpyrrolidone (PVP)-40, and 7% (v/v) 2-mercaptoethanol.

^(b)^ PCI: phenol (pH 4.3): chloroform: isoamyl alcohol (1.0: 0.8: 0.2).

**Table 2:** Comparison of the TENS-PCI method with other RNA isolation procedures used for plant species with high levels of secondary metabolites.

Method	RNA yield ^(a)^ (*μ*g/mg tissue)			RNA purity ^(a)^	
					Leaf	
	Leaf	Stem	Root	A_260_ : A_230_	A_260_ : A_280_
*Sugarcane (*Saccharum *spp. hybrid) (present study) *					
TENS-PCI: Micro-scale	0.59 ± 0.07	0.44 ± 0.05	0.45 ± 0.03	1.99 ± 0.07	1.97 ± 0.09
Macro-scale	2.42 ± 0.15	3.92 ± 0.27	0.99 ± 0.08	2.01 ± 0.07	1.98 ± 0.06
RNeasy Plant Mini Kit					
Guanidine isothiocyanate	0.10 ± 0.04	NA ^(b)^	NA	2.30 ± 0.08	1.95 ± 0.10
Guanidine hydrochloride	0.11 ± 0.04	NA	NA	1.20 ± 0.06	1.92 ± 0.11
FastRNA Pro Green Kit (Phenol-based)	0.11 ± 0.03	NA	NA	1.17 ± 0.05	1.79 ± 0.07
*Sweet orange (*Citrus sinensis* Pers.) (present study) *					
TENS-PCI: Macro-scale	2.29 ± 0.11	NA	NA	2.13 ± 0.01	2.07 ± 0.01
*Woody plants*					
(1) Grapevine (*Vitis* spp.) [44]					
Rapid CTAB	0.11 ± 0.05	0.11 ± 0.03	NA	2.29 ± 0.12	1.89 ± 0.03
RNeasy kit	0.04 ± 0.01	0.04 ± 0.01	NA	0.36 ± 0.05	1.07 ± 0.04
Guanidine	0.12 ± 0.02	0.10 ± 0.02	NA	1.11 ± 0.08	1.77 ± 0.10
(2) Lemon tree (*Citrus limon *Burm.f.)	0.14 ± 0.03	0.06 ± 0.01	NA	2.28 ± 0.14	2.01 ± 0.03
Rapid CTAB [44]					
(3) Plum (*Prunus domestica* L.)	0.18 ± 0.01	0.21 ± 0.04	NA	2.23 ± 0.11	2.03 ± 0.02
Rapid CTAB [44]					
(4) London plane tree (*Platanus acerifolia *Willd.) [6]					
Guanidine	0.20 ± 0.004	NA	NA	1.49 ± 0.07	1.35 ± 0.11
SDS/phenol	0.12 ± 0.004	NA	NA	1.47 ± 0.06	1.49 ± 0.89
CTAB	0.26 ± 0.007	NA	NA	1.61 ± 0.07	1.62 ± 0.79

^(a)^Data represent mean and standard error of at least 6 biological replicates and 6 technical repeats.

^(b)^NA: data not available.

**Table 3:** Relative mRNA levels of representative defense- and stress-responsive genes that are two-fold up-regulated in sugarcane after treatment with the pathogen *Sorghum mosaic virus* (SrMV) or the stress-regulated hormones, salicylic acid (SA) and methyl jasmonate (MeJA), as detected by microarray and qRT-PCR analyses.

Fold induction or repression											
Putative function	Accession no.	Stem/Leaf	SrMV	SA (h)				MeJA (h)	
				Microarray		qRT-PCR		Microarray		qRT-PCR	
				24	48	24	48	24	48	24	48
(1) Genes coding for antimicrobial proteins											
*Chitinase *	AW746272	1.48	0.98	1.2	**9.28**	1.82	**12.36**	1.27	0.31	1.87	1.56
(2) Genes coding for proteins involved in secondary metabolism											
*O* *-methyltransferase *	NM_001155649	**3.2**	1.03	0.67	**2**	1.01	3.52	**3.99**	**2.53**	**2.53**	0.3
* Dirigent*-*SoDIR1 *	AY421731										
DIR1		**2.4**	1.1	1.23	**17.21**	1.84	**32.41**	1.68	0.62	1.99	1.18
DIR3		**2.54**	1.02	1.24	**9.09**	1.79	**14.05**	**2.25**	0.34	**4.26**	1.75
DIR8		**2.18**	0.98	0.99	**5.27**	1.89	**8.89**	1.75	0.64	1.96	1.61
DIR9		**2.3**	1.11	1.42	1.02	1.97	1.97	**3.05**	0.79	**7.98**	1.79
DIR10		**3.58**	0.96	1.43	**21.66**	**2.56**	**30.33**	**2.17**	0.29	**3.65**	0.97
DIR27		**2.62**	1.1	0.97	**6.93**	1.75	**10.06**	1.42	0.54	1.98	1.36
DIR32		**2.96**	1.03	1.16	**7.73**	1.99	**11.04**	1.14	0.43	1.96	1.06
DIR34		**2.3**	1.04	1.4	0.7	1.88	1.09	**3.08**	1.06	**6.27**	1.97
DIR44		**7.84**	0.89	1.24	**19.22**	1.94	**29.18**	**4**	0.79	**9.03**	1.74
											
*Dirigent*-*SoDIR2 *	AJ626722										
DIR11		**4.83**	0.91	1.33	**20.96**	1.08	**5.3**	**6.45**	1.98	**7.21**	**2.23**
DIR37		**2.62**	1.01	1.04	**11.32**	**2.12**	**19.24**	1.32	0.35	1.98	0.88
											
*Dirigent-like *	AY781903										
DIR12		**3.97**	0.99	1.01	**15.81**	1.97	**26.09**	**3.16**	0.2	**6.04**	1.45
DIR13		**3.25**	1.13	1.25	**18.19**	**2.02**	**30.56**	**2.25**	0.24	**5.12**	1.87
DIR16		**3.08**	0.94	1.01	**19.64**	**2.02**	**19.86**	**8.67**	1.16	**9.97**	**2.99**
											

Relative abundance of mRNA transcripts of the cDNAs was determined in sugarcane stem, leaf, and root as well as in stem of plants infected with SrMV or treated with SA or MeJA. Values of each transcript were normalized to that of the sugarcane constitutive *ubiquitin* gene. Values represent the average normalized ratios of transcripts obtained: (1) from stems to those obtained from leaves, and (2) from stems at the indicated time of treatment to those obtained from the untreated (0 time or no infection). Data are representative of two biological samples and three technical repetitions. Values for cDNAs that are two-fold up-regulated are in bold.
