# Supplementary material for: Reproducible RNA Preparation from Sugarcane and Citrus for Functional Genomic Applications
Source: Int J Plant Genomics. 2010 Jan 27;2009:765367. doi: 10.1155/2009/765367 (PMC2817868; doi:10.1155/2009/765367)
Supplement: Supplementary file 1 — Supplementary FIGURE 1S: Reproducibility of the data obtained from the microarray analysis of gene expression profiling of the sugarcane stem, using amplified RNA (aRNA). [file 765367.f1.pdf]

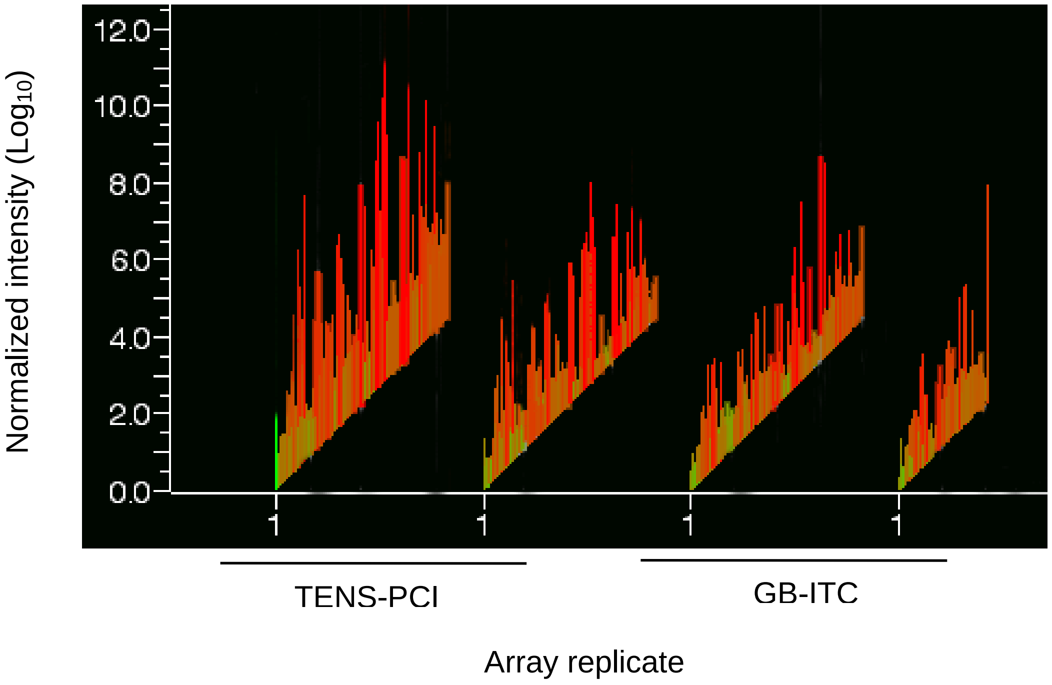


Supplementary Figure 1S: Reproducibility of the data obtained from the microarray analysis of gene expression profiling of the sugarcane stem, using amplified RNA (aRNA). A representative three-dimensional graph is shown that compares the normalized $Log_2$ gene expression from two independent replicate “stem versus leaf tissue” hybridizations using aRNA derived from the TENS-PCI method or the RNeasy-guanidine isothiocyanate (GB-ITC) method. Changes in gene expression are color-coded; red represents up-regulation in stem and green represents down-regulation in stem. The graph was generated using GeneSpring software (Agilent Technologies, Palo Alto, CA).
